# Renal microvascular lesions in lupus nephritis

**DOI:** 10.1080/0886022X.2019.1702057

**Published:** 2019-12-20

**Authors:** Ying Ding, Ying Tan, Zhen Qu, Feng Yu

**Affiliations:** a Department of Nephrology, Peking University International Hospital, Beijing, PR. China; b Renal Division, Department of Medicine, Peking University First Hospital, Institute of Nephrology, Peking University, Beijing, PR. China; c Key laboratory of Renal Disease, Ministry of Health of China, Beijing, PR. China; d Key Laboratory of CKD Prevention and Treatment, Ministry of Education of China, Beijing, PR. China

**Keywords:** Renal microvascular lesions, lupus nephritis, immunological injury, thrombosis

## Abstract

Renal microvascular lesions, common in lupus nephritis (LN), are associated with long-term poor outcomes. There are mainly five pathological types of renal microvascular lesions in LN: (1) vascular immune complex deposits (ICD), (2) arteriosclerosis (AS), (3) thrombotic microangiopathy (TMA), (4) non-inflammatory necrotizing vasculopathy (NNV), and (5) true renal vasculitis (TRV). The pathogenesis of renal microvascular lesions in LN remains to be elucidated. The activation and dysfunction of endothelial cells, in addition to the contribution of immune system dysfunction, especially the immune complex-induced vascular inflammation and antiphospholipid antibody-associated thrombotic events, are key mechanisms in the development of vascular lesions in LN that need to be further investigated. Alteration of the microvascular environment produces an acute immunological response that recruits immune cells, such as T cells, monocytes, and macrophages, which induces platelet aggregation with microthrombus formation. There is also increased cytotoxicity caused by cytokines produced by immune cells in the kidney. Identifying the mechanism underlying the pathogenesis of renal microvascular lesions in LN might provide potential targets for the development of novel therapies.

## Introduction

Lupus nephritis (LN) is one of the most common and severe manifestations of systemic lupus erythematosus (SLE). The renal histopathological lesions are closely associated with different clinical characteristics, therapeutic responses, and outcomes in LN patients [1], and can present as glomerular, tubulo-interstitial, and micro-vascular lesions. Renal microvascular lesions are common in LN and are becoming increasingly recognized as a hallmark of the disease. In addition to glomerulonephritis, there is accumulating evidence showing that renal vascular lesions can adversely affect the long-term renal outcomes and might play an important role in the therapeutic strategy choice [2,3]. The 2003 International Society of Nephrology/Renal Pathology Society (ISN/RPS) specified the importance of vascular damage: ‘Indicate and grade tubular atrophy, interstitial inflammation and fibrosis, severity of arteriosclerosis or other vascular lesions’ [4]. The consensus report published in 2018 derived from a meeting of 18 members of an international nephropathology working group in Leiden further proposed that vascular lesions should be evaluated, and a grading system should be created [5].

The pathogenesis of renal microvascular lesions in LN is not fully understood. Results from animal models and human lupus experiments have suggested that the activation and dysfunction of endothelial cells, in addition to the contribution of immune system dysfunction, especially the immune complex-induced vascular inflammation and antiphospholipid antibody-associated thrombotic events, are key mechanisms in the development of renal microvascular lesions in LN that need to be further investigated [6]. Although different types of vascular lesions share certain common mechanisms, each morphological lesion type may exhibit its own unique factors that result in a morphological presentation that differs from other types of lesions. In this review, clinical characteristics of various renal microvascular lesions in LN are described, and the underlying pathogenesis and optimization strategies for patients with LN are further discussed.

## Classification and clinical presentations of renal microvascular lesions

**Figure 1.**
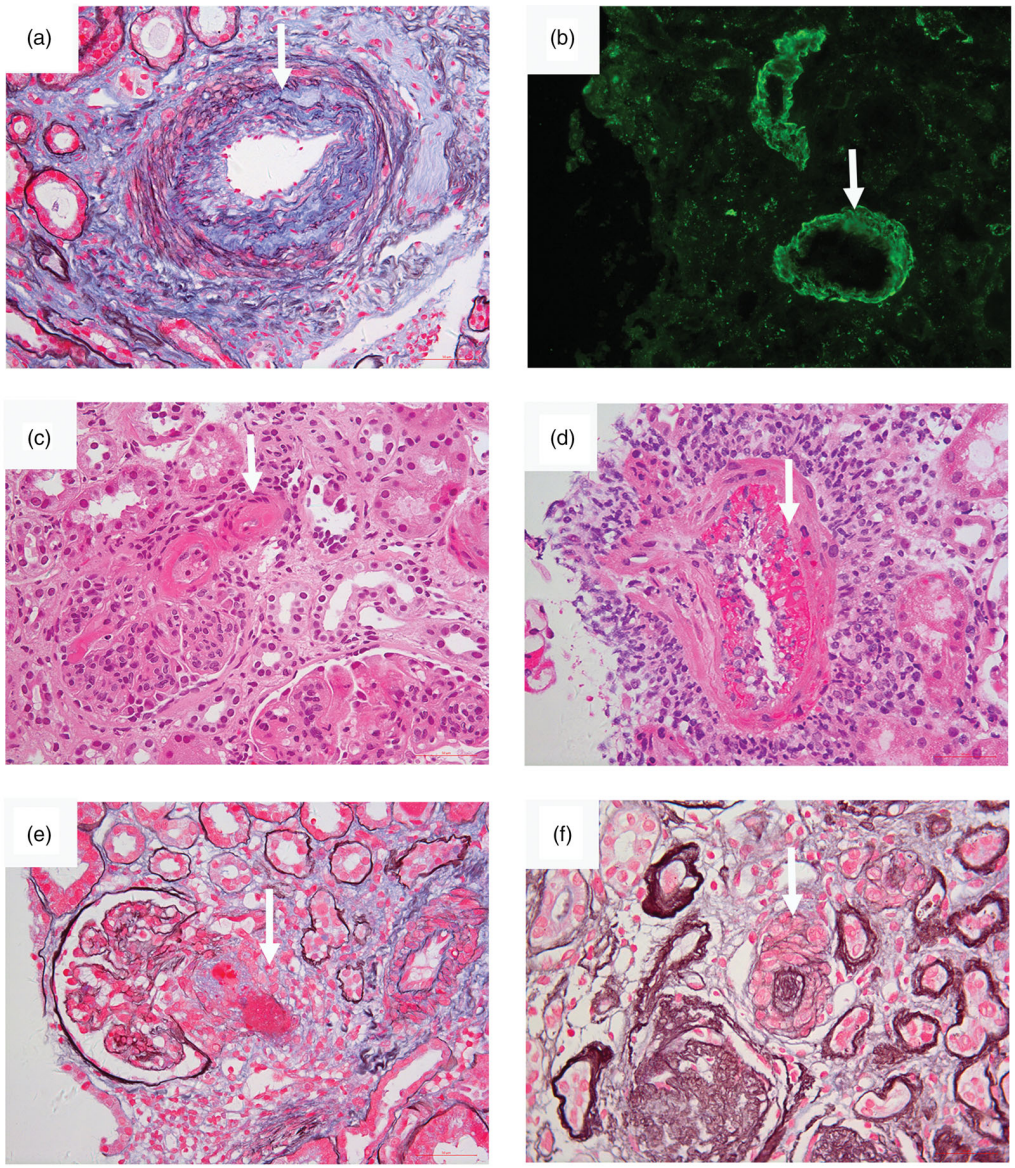
Photomicrographs of various renal microvascular lesions in lupus nephritis. (a) Arteriosclerosis. Fibrous proliferation of the intima of an interlobular arteriole is seen (arrow) (periodic acid-silver methenamine and Masson’s trichrome, original magnification × 400). (b) Immune complex deposits. By immunofluorescence, deposits of IgG were detected (arrow) in the walls of interlobular arteriole (IF, original magnification ×200). (c) Noninflammatory necrotizing vasculopathy. Deposits of glassy eosinophilic material was deposited (arrow) in the vessel wall. There was no inflammatory infiltration in the vessel wall (hematoxylin and eosin, original magnification ×400). (d) True renal vasculitis. Fibrinoid necrotizing vasculitis was present (arrow) in the vessel (hematoxylin and eosin, original magnification ×400). (e) Thrombotic microangiopathy. Thrombosis (arrow) was seen in an afferent arteriole (periodic acid-silver methenamine and Masson’s trichrome, original magnification, original magnification ×400). (f) Thrombotic microangiopathy. “Onion skin” pattern lesion (arrow) was seen in the vessel (periodic acid-silver methenamine, original magnification ×400).

Appel et al. and D’Agati provided a pathological classification for lupus microvascular lesions (Table 1) [1,7]. There are five common pathological types of renal microvascular lesions in LN, including vascular immune complex deposits (ICD), arteriosclerosis (AS), thrombotic microangiopathy (TMA), non-inflammatory necrotizing vasculopathy (NNV), and true renal vasculitis (TRV). The typical images of each renal microvascular lesions (From renal pathological center in Peking University First Hospital) were presented in Figure 1. Lupus microvascular lesions is relatively common, and its prevalence in LN varied from 53.4% to 81.8% among different healthcare centers [2,3,8]. It should be noted that there might exist some links between renal vascular lesions and extra-renal vascular lesions like CNS.

**Table 1.** Pathological classification of renal microvascular lesions in LN [2,7].

Classification	Pathological descriptions
Arteriosclerosis (AS)	“Arteriosclerosis” or “arterial intimal fibrosis” denotes thickening of the arterial wall and narrowing of the vascular lumen produced by fibrotic intimal thickening and replication of the internal elastic lamina.
Vascular immune complex deposits (ICD)	The extra-glomerular arteries and arterioles with ICD usually appear normal by light microscopy, without signs of necrosis, thrombosis, or inflammatory infiltration of the blood vessel walls. IgG, IgA, IgM, and various complement components can be found in the vessel wall by immunofluorescence microscopy. The discrete electron-dense deposits are most commonly seen beneath the vascular endothelium or within the basement membranes by electron microscopy.
Thrombotic microangiopathy (TMA)	During the early stages, arterioles show swelling of the endothelial cells and subendothelial space. The arteriolar lumen may be severely narrowed, and fibrinoid necrosis may occur. Interlobular renal arteries may show swelling of the intima, which may be accompanied by mucoid intimal hyperplasia. The cellular intimal proliferation may be seen and give rise to an “onion skin” pattern lesion during the course of the disease. By immunofluorescence microscopy, arterioles and small arteries often exhibit fibrinogen or fibrin in their walls, usually in a subendothelial position. IgM positivity can be detected in vessel walls, as well as C3, C1q, IgG, and IgA. Intravascular thrombi also show positive fluorescence for fibrinogen or fibrin. By electron microscopy, swelling and detachment of the endothelium from the underlying structures with a widening of the intima can be seen. Structures consistent with fibrin can be found at the vessel wall. During the later stages, elongated myointimal cells abound in the thickened intima.
Non-inflammatory necrotizing vasculopathy (NNV)	By light microscopy,the affected vessels are severely narrowed and sometimes occluded by abundant intimal and luminal deposits of glassy eosinophilic material that may extend into the media. The endothelium is often swollen or denuded, and the elastic membrane of the interlobular arteries is often disrupted. There is smudgy degeneration and loss of medial myocyte, while the inflammatory infiltrate is rare with a few lymphocytes in the lumen or intima occasionally. By immunofluorescence microscopy, various staining patterns for IgG, IgM, IgA, complement components, and fibrin-related antigens are typically present in the blood vessel walls. By electron microscopy, massive confluent intraluminal and mural deposits of granular electron-dense material can be detected with swelling or loss of endothelium.
True renal vasculitis (TRV)	By light microscopy, there is true inflammatory infiltration of the intima and media by neutrophils and mononuclear leukocytes, often accompanied by fibrinoid necrosis and rupture of elastic lamellae. Immunofluorescence discloses staining for fibrin-related antigens, with weak and more variable staining for immunoglobulin and complement.

C3: complement component 3; C1q: complement component 1q.

The data from our center showed that among 341 patients with pathologically diagnosed LN, 279 (81.8%) patients with renal vascular lesions were identified, including 74.2% with ICD, 24.0% with AS, 17.6% with TMA, 3.8% with NNV, and 0.6% with TRV. A total of 105 patients presented with more than two types of vascular lesions [2].

A study of 161 renal biopsies with SLE selected from the data registry at the University of Toronto Lupus Clinic (UTLC) revealed the existence of renal vascular lesions in 75.2% of the patients, in which AS was the most frequent lesion, occurring in 57.8% patients, followed by TMA (8.1%), uncomplicated vascular immune deposits (6.2%), NNV (3.1%), and TRV (0%) [3]. Another single-center study composed of 429 biopsy-proven LN patients showed the presence of renal vasculopathy in 53.4% of the patients, including AS (44.0%), NNV (1.4%), TMA (5.4%), and TRV (2.6%) [8].

The clinical presentations of patients with and without renal microvascular involvement was different. Data from our center showed the proportions of hypertension, anemia, hematuria, and acute renal failure and the scores of Systemic Lupus Erythematosus Disease Activity Index (SLEDAI) to be significantly different among groups (included the group of ICD, TMA, AS, ICD + AS and NRVL), which was the highest in TMA subgroup. Besides we scored the renal microvascular lesions in SLE patients and analyzed their correlations with glomerular and tubulointerstitial lesions in the NIH evaluation system. We found that the scores of microvascular lesions were positively correlated with the scores of total AIs (*r* = 0.26, *p* < 0.001), endocapillary hypercellularity (*r* = 0.22, *p* < 0.001), cellular crescents (*r* = 0.22, *p* < 0.001), interstitial inflammatory cell infiltration (*r* = 0.25, *p* < 0.001), total CIs (*r* = 0.27, *p* < 0.001), tubular atrophy (*r* = 0.25, *p* < 0.001), and interstitial fibrosis (*r* = 0.22, *p* < 0.001). The prevalence of positive anti-dsDNA antibodies was significantly higher in patients with vascular immune complex deposits and TMA compared with other groups (*p* < 0.001) [2]. Study from Mejia-Vilet et al. indicated that the patients without vascular lesions (NVL) were younger and presented with a better renal function and a lower blood pressure, in which patients with TMA tended to have the worst clinical manifestations with the highest level of serum creatinine value and mean arterial pressure. They also found that the renal TMA patients had a higher prevalence of positive lupus anticoagulant serology and APS diagnosis [8].

The prognosis of different renal microvascular lesions was different. Data from our center showed that the mortality rates among them were not different, but the renal survival rates were significantly different between the groups with and without renal vascular lesions (*p* = 0.005), in which TMA group showed the poorest renal outcome, ICD being the second (average follow-up time of 5 years) [2]. Recent data from a single tertiary-care center in Mexico City showed that 5-year renal survival was 83% for NVL, 63% for AS, 67% for NNV, 31% for TMA and 33% for TRV [8].

## Pathogenesis of vascular lesions in LN

**Figure 2.**
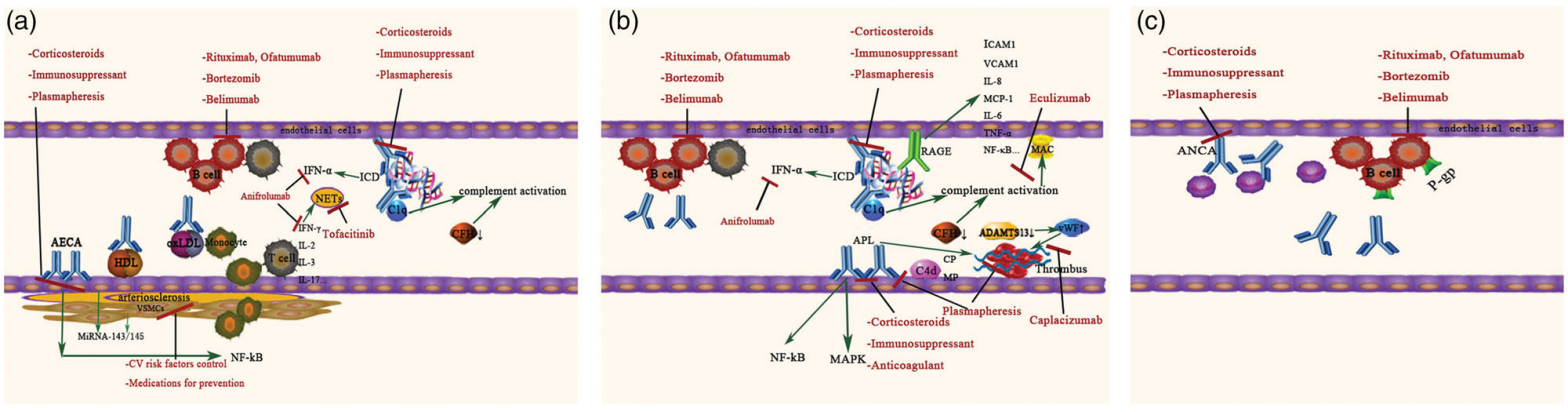
Pathogenic mechanisms and potential therapeutic targets for renal microvascular lesions in lupus nephritis. (a) Potential pathogenesis and treatment involved in arteriosclerosis (AS). Various autoantibodies such as anti-endothelial cell antibodies (AECA), anti-oxidized LDL antibodies and anti-HDL antibodies play important roles in the pathogenesis of AS by inducing a proinflammatory endothelial cell phenotype and interfering with lipoprotein metabolism *via* activation of NF-κB pathway, which contribute to the formation of AS. Immune complex deposits and complement system were also involved in the pathogenesis of AS. T cells expressing proinflammatory cytokines, such as interferon-γ (IFN-γ), which favor neutrophil extracellular trap (NET) formation, might play a role in the development of arteriosclerosis lesions. Potential treatment: Corticosteroids and immunosuppressants are classical treatments, which could be the “baseline therapy” for renal microvascular lesions. Plasmapheresis and immunomodulating treatment targeting B-cells and plasmocytes could be used to eliminate the pathogenic autoantibodies. Cytokines blockers, such as tofacitinib and anifrolumab, could prevent type I IFN responses and NET formation. Cardiovascular risk factors prevention, including renin-angiotensin system inhibitors and statin, may play a role in preventing arteriosclerosis. (b) Potential pathogenesis and treatment involved in immune complex deposits (ICD), thrombotic microangiopathy (TMA) and non-inflammatory necrotic vasculopathy (NNV). Immune complexes (ICs) elicit proinflammatory responses in human endothelial cells and alter their function *via* the high-mobility group box 1 protein (HMGB1)–receptor for advanced glycation end-products (RAGE) axis. Besides, ICs could serve as endogenous IFN-α inducers, stimulating the production of IFN-α, together with other cytokines, contributing to the formation of immune complex deposits (ICD) lesions. Complement activation, deficiency of A disintegrin-like and metalloproteinase with a thrombospondin type 1 motif 13 (ADAMTS-13) activity leading to overexpression of large von Willebrand factor (vWF), together with the antiphospholipid antibodies (aPLs) activating endothelial cells, platelets and monocytes through nuclear factor-κB (NF-κB) and mitogen-activated protein kinases (MAPKs) pathway, resulting in the formation of TMA lesions. Non-inflammatory necrotic vasculopathy (NNV) lesions might share similar pathogenesis as ICD lesions since it was found to be always co-present with ICD lesion. Potential treatment: Corticosteroids and immunosuppressants are classical treatments. Anticoagulation and plasmapheresis are recommended for both antiphospholipid syndrome nephropathy (APSN) and thrombotic thrombocytopenia purpura (TTP). Inhibitors of the complement system, such as eculizumab, might have therapeutic value in TMA. Caplacizumab, which blocks vWF activity, is a promising therapy for TTP. Immunomodulating treatment targeting B-cells and plasmocytes could attenuate the production of pathological antibodies. Cytokines blockers, such as anifrolumab, could prevent type I IFN responses. (c) Potential pathogenesis and treatment involved in true renal vasculitis (TRV). Anti-neutrophil cytoplasmic autoantibodies (ANCAs) and accumulation of P-gp-overexpressing B cells at site might play a role in its pathogenesis. Potential treatment: Corticosteroids, immunosuppressants, and immunomodulating treatment targeting B-cells and plasmocytes could be the potential treatment.

Since up to one-third of the LN patients presented simultaneously with more than two types of vascular lesions, it would be expected that there would be some common pathogenic pathways involved in all types of vascular lesions, although each lesion may also have its own unique mechanism that causes a characteristic presentation distinct from the others (Figure 2). The exact initial event in renal microvascular lesion in SLE remained to be elucidated. Considering previous works in literature, we proposed that the dysregulation of immune system might be the initial event which could result in the dysfunction/activation of endothelium. SLE is characterized by the loss of immunological tolerance with the subsequent autoantibodies production. Immune system dysregulation resulting in the formation of autoantibodies and the succedent immune complex formation, resulting in the injury/activation of endothelial cells and recruitment of immune cells which triggers an inflammatory response involving activation of complement cascade, increasing expression of adhesion molecules and the production of inflammatory cytokines and chemokines, thus inducing clinico-pathological vascular changes [6]. It is also noted that the genetic, epigenetic, environmental, hormonal factors which are involved in the activation and dysfunction of the endothelial cells in which endothelium of those patients are prone to be activated or injured in the second hit should be considered. This section reviewed the five pathological types of renal vascular lesions in LN.

### Arteriosclerosis (AS)

Atherosclerosis is the most common subtype of arteriosclerosis, which is the term used in most of the studies concerning vasculopathy in LN [2,9]. Chronic inflammation is considered to be the hallmark of atherosclerosis, and inflammatory processes are instrumental during all stages of the progression of atherosclerosis [10]. Autoantibodies triggering endothelial injury and dysfunction seem to be the initial step in atherogenesis, together with the impaired clearance of immune complexes (ICs), complement activation, cytokine-mediated damage, participation of immunocytes, and epigenetic alterations.

Various autoantibodies in LN were shown to affect endothelial cells and cause chronic vessel wall damage [9]. Anti-endothelial cell antibodies (AECA) represent a heterogeneous family of autoantibodies directed against structural endothelial proteins and can be detected in SLE patients, which can induce a proinflammatory and pro-adhesive endothelial cell phenotype *via* activation of the nuclear factor κB (NF-κB) transcription factor pathway with subsequent increased monocyte adhesion [11,12]. Antibodies to oxidized low-density lipoprotein (anti-oxLDL) facilitate foam cell generation and increase with the anti-double-strand DNA (ds-DNA) antibody titer, complement activation, and disease activity scores in SLE patients [13,14]. High-density lipoprotein (HDL) plays an important role in preventing the oxidation of LDL and its consequent uptake by monocytes, thus preventing the formation of foam cells which was one of the most important steps in atherogenesis. Antibodies to high-density lipoprotein (HDL) were also found in SLE patients, which contributed to endothelial cell dysfunction by favoring the oxidation of LDL [15]. These antibodies might contribute to the pathogenesis of atherosclerosis by causing injury to the endothelium and altering the metabolism of lipoproteins involved in atherogenesis.

More than one-third of the lupus patients with renal atherosclerosis were combined with ICD [16], suggesting its involvement in this change formation. A deficiency of complement factors in both the classic and lectin pathway, including complement component 1q (C1q), mannose-binding lection (MBL), and complement component 3 (C3), in a low-density lipoprotein receptor-deficient (*Ldlr*−/−) murine model of lupus has been shown to result in larger atherosclerotic lesions. Thus, a lower level of complement factors may block the sweeper role of the complement system and serve as an important underlying mechanism for the increased levels of plaque [17–19].

The importance of the role of cytokine-mediated endothelial damage was also investigated. Immunohistochemistry of renal biopsies from patients with LN confirmed that IFN-α promoted an antiangiogenic signature through repression of vascular endothelial growth factor (VEGF)-A and induction of an IL-1R antagonist, which correlated with decreased renal vascular density and vascular rarefaction, interfering with vascular repair in SLE [20]. IFN-β may promote atherosclerosis by promoting macrophage recruitment to arteries [21]. IFN-γ is the prototypical Th1 cytokine, which can promote plaque instability, foam cell formation, and plaque rupture [22]. In addition, the interplay between type I IFNs and aberrant neutrophil function to form neutrophil extracellular traps (NETs) could further propagate immune dysregulation and endothelial damage in SLE [23]. Peptidyl arginine deiminase (PAD) inhibition which can reduce NET formation was proved the ability to protect against kidney and lupus-related vascular damage in New Zealand Mixed model of lupus [24].

Epigenetic alterations might also contribute to the pathogenesis of AS lesions during SLE. Recent studies have revealed that miRNA-145 expression was mainly detected in renal vascular smooth muscle cells and epithelial cells and was associated with vascular injury in LN patients [25].

### Immune complex deposits (ICD)

Accumulation of immunoglobulins (Igs) and complement components in the vascular wall strongly support the role of immunologic injury in the development of renal vascular lesions during SLE. ICs in SLE are biological complexes, which typically contain autoantigens nucleic acids, nucleic acids-associated proteins, and corresponding autoantibodies [26]. Previous studies have reported that effective components of ICs include endogenous nucleic acids Class A CpG oligodeoxynucleotides (CpG-A), high-mobility group box 1 protein (HMGB1), and lupus IgG [26–28]. HMGB1, a nuclear DNA-binding protein released from necrotic cells, is an essential component of DNA-containing ICs. ICs upregulate the cell-surface expression of receptor for advanced glycation end products (RAGE) *via* HMGB1, the expression of intercellular adhesion molecule-1 (ICAM-1) and vascular cell adhesion molecule-1 (VCAM-1), as well as increase the secretion of a series of chemokines and promote the activation of the transcription factor NF-κB p65 in human endothelial cells, thus increasing the migration of monocytes across the human endothelium monolayer to inflammatory sites. Moreover, a previous study showed that HMGB1 and CpG-A, which are capable of inducing responses in human endothelial cells, exhibited a synergistic effect when used together as compared to when used alone [27].

Natural IFN-α producing cells (NIPC) and plasmacytoid dendritic cells (pDC) can be stimulated by microorganisms and different viruses [29]. ICs containing DNA/RNA and IgG with anti-DNA/RNA specificity serve as endogenous IFN-α inducers that have been shown to mimic the role of exogenous inducers and continuously stimulate the production of IFN-α in the NIPC and pDC in SLE [30]. The IFN-α-inducing activity in the case of apoptotic cells and SLE-IgG was dependent upon the Fc portion of the SLE-IgG [31]. ICs inducing IFN-α might destroy the balance between vascular damage and repair, thus resulting in the progression of vasculopathy during LN.

IgG is considered to be the predominant antibody constituting the ICDs in LN [32]. It was thought that the pathology underlying this type of vasculopathy was similar to those that mediate glomerular and tubulo-interstitial immune deposits formation. However, Satoskar et al. found that in a total of 40 arterial/arteriolar IgG-positive patients, the IgG subclass pattern was discordant in 27 biopsies, with stronger IgG subclass staining in the vascular walls of six patients. This indicated that the IgG subclass may not just represent the same circulating ICs distributed within the different compartments of the kidney. They also found that the staining for each of the IgG subclasses showed better correlation with C1q than with C3 in the glomeruli, tubular basement membrane (TBM), and vasculature, which supported the role of classical complement pathway activation in the development of tissue injury in LN [33]. The ICs in the vascular endothelium trigger an inflammatory response, which involves activation of the complement cascade and subsequent destruction of the vascular basal membranes and inflammatory cell infiltrates. The deficiency or dysfunction of some complement regulators, such as complement factor H (CFH), would induce the immunological attack or lose the protection from the injury [34].

### Thrombotic microangiopathy (TMA)

Renal TMA in LN is multifactorial and can be attributed to several factors, including the complement system, anti-phospholipid syndrome (APS), thrombotic thrombocytopenic purpura-hemolytic uremic syndrome (TTP-HUS), and drugs. In patients with renal TMA, long-term outcomes were poorer when they had both positive glomerular C4d deposition and lower serum CFH levels [35], which supports that activation of the complement system might be involved. Interestingly, Cohen et al. observed a striking relationship between the intensity of glomerular C4d deposition and the presence of renal microthrombi in LN. A significantly higher percentage of intense C4d staining was observed in a microthrombi group (7/8 patients) as compared to a non-detectable microthrombi group (8/30 patients) [36]. Another larger prospective cohort study of 155 patients with LN showed similar results with intense C4d staining in 13/28 patients in the glomerular microthrombosis group as compared to 20/127 patients in the non-glomerular microthrombosis group [37]. These findings indicated that complement activation might be involved in the induction of thrombosis. Another study in patients with TMA with a variety of clinical conditions revealed that renal C4d was a common denominator in TMA, regardless of the underlying clinical conditions, with a predominance of classical pathway activation (90.5%) [38].

Antiphospholipid antibodies (aPLs) associated thrombotic events are important mechanisms of renal TMA in SLE. aPLs were positive in 30–40% of SLE patients [39]. SLE patients with aPLs have a higher prevalence of thrombosis, pregnancy morbidity, pulmonary hypertension, livedo reticularis, acute/chronic renal vascular lesions and neuropsychiatric involvement compared to those without aPLs. aPLs do not cause thrombosis directly, but rather induce a clotting-prone context (called a “first hit”) in which thrombotic events are triggered by additional insults (called a “second hit”). Currently, two main molecular mechanisms have been proposed to explain the “two hits” theory by which aPLs induce a pro-coagulant and pro-adhesive state. First, aPLs can regulate clotting and fibrinolytic pathways by interacting with proteins [40]; aPLs can prevent inactivation of coagulation factors by inhibition of natural anticoagulants such as antithrombin 3, protein C, and annexin A5 [40,41]; and aPLs were also shown to impair fibrinolysis [13,42]. Second, aPLs can activate endothelial cells, platelets, and monocytes through the interaction with membrane-bound proteins and receptors through NF-κB and mitogen-activated protein kinases (MAPKs) pathway. These signaling events induce pro-inflammatory and pro-coagulant phenotypic changes, expression of adhesion molecules, and the release of tissue factor and fibrinolysis inhibitors [40,42].

A disintegrin-like and metalloproteinase with a thrombospondin type 1 motif 13 (ADAMTS-13), which has powerful and natural anti-thrombotic activity, was confirmed to contribute to renal TMA in LN. Our previous study demonstrated that serum ADAMTS-13 activity was significantly lower in patients with both LN and TTP-HUS than in patients with LN only (40% versus 69%, *p* = 0.012) and in normal control (40% versus 81%, *p* < 0.001). After clinical remission, the serum ADAMTS-13 activity of the 6 patients with TTP-HUS increased significantly [43]. Additionally, Tati et al. found that the microvascular process induced by ADAMTS-13 deficiency could trigger complement activation in platelets and the endothelium *in vitro*, which might influence the development of TMA-like changes [44].

### Non-inflammatory necrotic vasculopathy (NNV)

NNV, which could also be called as hyaline thrombi, lupus vasculopathy and renal angiitis [45], is a relatively rare form of renal microvascular lesions, which has not been found as an isolated form in any individual. The different terminologies in use reflected the uncertainly to its pathogenesis. However, vascular ICDs were found to be co-present in nearly all of the patient biopsies, which raised the hypothesis that NNV might be a transition form from vascular ICDs to other severe vascular lesions, such as TMA [2,7]. The fact that most NNV patients were found to have active proliferative nephritis in the presence of abundant ICDs strongly supported a role of immunologic factors in the development of the lesions. Endothelial injury initiated by the deposits of ICs further induced the lesions of NNV [7]. However, the exact cytokine or adhesion molecules involved in NNV remain to be revealed.

### True renal vasculitis (TRV)

TRV is the least frequent renal vasculopathy encountered in LN and has been infrequently reported in the literature. Anti-neutrophil cytoplasmic autoantibodies (ANCAs) have been reported to be in 15–20% of SLE patients with P-ANCA as the dominant type [46]. TRV and ANCA-associated systemic vasculitis share similar pathological features in which IgGs, complement components, and electron-dense deposits are virtually not seen, hinting at the role of ANCA in the development of TRV in LN.

While not all SLE patients with TRV are ANCA positive [47], other mechanisms remain to be elucidated. The involvement of autoantibodies in the pathogenesis of TRV potentially indicates the involvement of B cells. P-glycoprotein (P-gp) is a member of the ATP-binding cassette transporter superfamily and functions as an energy-dependent transmembrane efflux pump. Accumulation of P-gp-overexpressing B cells was found at the site of TRV in a patient with minimal change LN, which might induce antibodies favoring the formation of ICs, which directly infiltrate into the perivascular lesions of small vessels, leading to the formation of TRV [48].

## Therapeutic strategies for renal microvascular lesions in LN

The therapeutic intervention for renal microvascular lesions in LN remains unclear, and the current treatment protocol is mainly based on the glomerular pathology. Kidney Disease Improving Global Outcomes (KDIGO) guidelines only propose recommendations for TMA lesions involving APS nephropathy (APSN) and TTP, in which anticoagulation and plasma exchange were suggested separately [49]. Target therapies are still developing based on possible mechanisms.

The rationale for the use of plasmapheresis for vascular injury during LN is supported by the literature. Plasmapheresis has the potential to remove multiple plasma ICs, such as autoantibodies, abnormal ICs, and circulating protein-bound toxic agents. It is also able to administrate higher volumes of plasma and replace deficient or defective bio-functional proteins, such as coagulation factors and deficient complement factors. Our recent retrospective study focused on the use of plasmapheresis in LN patients with renal TMA. In comparison with the patients who only received conventional combined corticosteroids and immunosuppressive agents (nine clinically matched patients), the patients who received added plasmapheresis (nine patients) had a significantly higher rate of remission (77.8% versus 11.1%, *p* = 0.018) and less composite endpoints (*p* = 0.005), in which two patients reached ESRD in the plasmapheresis group, and 1 patient was dead and seven patients reached ESRD in the nonplasmapheresis group [50].

Given its important roles in the pathogenesis of vascular injury in LN, the complement system might be a potential therapeutic target. Eculizumab, a fully humanized monoclonal antibody that acts as a terminal complement inhibitor, targets the human C5 complement component, thereby preventing the generation of the membrane attack complex C5b-9 and the release of the C5a. It is the first therapy approved to treat paroxysmal nocturnal hemoglobinuria (PNH), atypical hemolytic uremic syndrome (aHUS) and severe Shiga toxin-associated hemolytic uremic syndrome [51–53]. Of note, eculizumab has been reported to induce successful treatment of lupus associated with TMA, APS, or severe proliferative LN in which conventional therapy, including plasmapheresis, failed [54–56]. A recent study reviewed 20 patients who received eculizumab with SLE and/or APS until 2017, where a hematological response was evident in 100% of the patients, and kidney recovery was evident in 85% of the patients. This study suggested that eculizumab might be a potential alternative treatment for patients with SLE and/or APS presenting with TMA and who were refractory to current immunosuppression therapies [57].

Since Von-Willebrand factor (vWF)-ADAMTS-13 system involved in the pathogenesis of renal microvascular lesions in LN, it could be a latent treatment target. Caplacizumab, a single-variable-domain Ig (Nanobody) directed to the A1 domain of vWF, was recently approved in the European Union for the treatment of acquired TTP (aTTP) in adults [58]. In an international phase III, double-blind, placebo-controlled trial, patients treated with caplacizumab (10 mg daily) exhibited a significant reduction in time-to-platelet count response as compared with placebo (platelet count normalization rate ratio 1.55 versus placebo *p* = 0.0099). Furthermore, a reduction in the recurrence and composite endpoint of TTP-related deaths was also observed in the caplacizumab group as compared to the placebo group (12.7% versus 49.3%, *p* < 0.001) [58,59]. Moreover, immunomodulating treatment, including monoclonal anti-CD20 antibody and proteasome inhibitors, was also used to treat patients with acute TTP or SLE or patients with TTP and SLE [60–64].

Immunocytes have a pivotal role in the pathogenesis of LN vasculopathy. Drugs targeting the immunocytes and their co-stimulating factors are currently being researched and developed. Belimumab, a human Ig-G1λ monoclonal antibody that inhibits soluble B lymphocytes stimulator (BlyS), has been approved for the treatment of extra-renal SLE [65] by the Food and Drug Administration (FDA) in the USA. Nevertheless, data from a phase 3 belimumab clinical trial showed that those who received belimumab treatment had a significantly lower and dose-dependent LN flare rate than placebo patients [66].

Inhibitors of cytokines or kinases, which are involved in the vascular damage of LN, may show new therapeutic opportunities. Tofacitinib is a Janus kinase (JAK) inhibitor that can modulate type I IFN responses and NET formation. Some studies have indicated that in *MRL/lpr* lupus prone mice, treatment with tofacitinib resulted in significant improvements in nephritis and endothelium-dependent vasorelaxation and endothelial differentiation [67]. Anifrolumab is a human IgG1k monoclonal antibody directed against subunit 1 of the type I IFN receptor, thus blocking the effects of type I IFNs. A phase II, randomized, double-blind, placebo-controlled study revealed that anifrolumab substantially reduced SLE disease activity compared with placebos across multiple clinical endpoints, especially in those with a high type I IFN signature at baseline. The role of anifrolumab in LN for the resolution of proteinuria in patients with proliferative LN is currently being investigated [68,69].

Our recent data have shown that in 79 patients with biopsy-proven LN, 50 had arteriosclerosis lesions on renal biopsy. Patients with arteriosclerosis presented with more severe echocardiographic indices, including a larger left atrial diameter, left ventricular end-diastolic diameter, and interventricular septum thickness as compared with those of patients without any vascular changes [16]. As patients with SLE experience greater risks from cardiovascular events compared to healthy matched controls, non-immunosuppressive therapies and modification of cardiovascular risk factors, including smoking cessation, glycemic control, and medications, such as renin-angiotensin system inhibitor and statin, should also be recommended for these patients [70,71]. Interestingly, a study investigating the effect of a therapy composed of an angiotensin converting enzyme inhibitors (ACEI) and/or statin in lupus-prone mice showed that mice treated with the combination therapy survived longer and showed a significant reduction in proteinuria, glomerular IgG deposit, and serum anti-DNA antibodies in comparison to the control mouse group. The mRNA expression of nephrogenic cytokines and chemokines, such as MCP-1, IL-4, and IFN-γ, was also found to be decreased in the ACEI/statin-treated group in this study [72]. The potential therapies for the treatment of renal microvasculopathy of LN are summarized in Table 2 and Figure 2.

**Table 2.** List of potential biological therapies for renal microvasculopathy in LN.

Drug Class	Commercial name	Evidence in lupus	Indications	Potential use
Anti-C5 mAb	Eculizumab	Clinical use	-PNH -aHUS	-Lupus associated TMA -APS -Severe proliferative LN
Anti-vWF	Caplacizumab	Phase III	/	-Acquired TTP
Anti-CD20 mAb	Rituximab (chimeric mAb)	Clinical use	-Lymphoma -refractory SLE and LN	-Lupus associated TTP
	Ofatumumab (fully humanized mAb)	Case report	/	-Refractory SLE and LN -Lupus associated TTP
Proteasome inhibitors	Bortezomib	Clinical trial	-multiple myeloma	-Refractory SLE and LN -Lupus associated TTP
Anti-BlyS	Belimumab	Phase III	-extra-renal SLE	-Lupus nephritis -Lupus associated TTP
JAK inhibitor	Tofacitinib	Phase Ib	-Rheumatoid arthritis (moderate to severe)	-Lupus nephritis -Lupus microvasculopathy
Anti-IFN I receptor	Anifrolumab	Phase IIb		-SLE -Proliferative LN -Lupus microvasculopathy

C5: complement component 5; mAb: monoclonal antibody; vWF: von-Willebrand factor; BlyS: B lymphocyte stimulator; JAK: Janus kinase; IFN I: interferon I; PNH: paroxysmal nocturnal hemoglobinuria; aHUS: atypical hemolytic uremic syndrome; SLE: systemic lupus erythematous; LN: lupus nephritis; TMA: thrombotic microangiopathy; APS: antiphospholipid syndrome; TTP: thrombotic thrombocytopenic purpura.

## Conclusions

Renal microvascular lesions are common in LN and are becoming increasingly recognized. The novelty of this article is that we deeply reviewed the renal microvascular changes in lupus nephritis based on potential pathophysiologic mechanism to clinical implications which might highlight the further explorations.
